# Ovarian Hyperstimulation Syndrome: A Simulation Case for Emergency Medicine Residents

**DOI:** 10.15766/mep_2374-8265.11271

**Published:** 2022-09-06

**Authors:** Kathleen A. Wittels, Katherine Dickerson Mayes, Andrew Eyre

**Affiliations:** 1 Assistant Professor, Department of Emergency Medicine, Brigham and Women's Hospital, Harvard Medical School; 2 Emergency Medicine Resident Physician, Department of Emergency Medicine, Brigham and Women's Hospital, Harvard Medical School; 3 Assistant Professor, Department of Emergency Medicine, STRATUS Center for Medical Simulation, Brigham and Women's Hospital, Harvard Medical School

**Keywords:** Ovarian Hyperstimulation Syndrome, Emergency Medicine, OB/GYN - Reproductive, Endocrinology/Infertility, Simulation

## Abstract

**Introduction:**

Ovarian hyperstimulation syndrome (OHSS) is a complication of assisted reproductive technology (ART) for infertility. Given the potential for significant morbidity, it is important for emergency medicine (EM) residents to be able to recognize and initiate treatment for this disorder.

**Methods:**

A high-fidelity human patient simulator was used, with availability of bedside ultrasound. PGY 1–4 EM residents participated in this case of a 28-year-old female patient undergoing treatment for infertility who presented to the emergency department with shortness of breath and near syncope. Workup revealed a diagnosis of OHSS. After the simulation, we surveyed residents on their knowledge of OHSS prior to participation in the simulation. We also asked about their confidence in caring for a patient with OHSS pre- and postsimulation based on a 5-point Likert scale.

**Results:**

A total of 24 EM residents completed this simulation case. Prior to participating in the simulation experience, 62% of residents reported that they had heard of OHSS, and 17% of residents had previously managed a patient with OHSS. After participating in the simulation, residents’ comfort with managing a patient with OHSS increased from 1.7 to 3.7 points (1 = *not at all comfortable,* 5 = *extremely comfortable*; *p* < .001).

**Discussion:**

OHSS is a rare but important complication of ART that many EM residents have not treated in the clinical environment. As the presenting symptoms may mimic other diagnoses, obtaining a detailed history and utilizing bedside ultrasonography are essential to diagnosing and correctly treating these patients.

## Educational Objectives

By the end of this activity, learners will be able to:

1. Verbalize the common signs and symptoms of ovarian hyperstimulation syndrome (OHSS).
2. Identify OHSS using common laboratory and radiologic testing.
3. Demonstrate appropriate supportive care for OHSS.

## Introduction

Infertility affects many women in the United States and worldwide, and an increasing number are utilizing assisted reproductive technology (ART) to assist with conception. Ovarian hyperstimulation syndrome (OHSS) is a potential complication of ART, affecting 20%–30% of patients undergoing ART.^1,2^ While many cases are mild, moderate and severe cases cause symptoms that can require emergency department evaluation.

Certain women are at higher risk for OHSS. Risk factors include younger age, history of polycystic ovarian syndrome, gonadotropin releasing hormone agonist cycles, and a high oocyte retrieval number.^1,3,4^ In addition, a woman who has previously had OHSS is at increased risk of a repeat occurrence.

Clinical manifestations of OHSS can be varied. Women with mild OHSS (i.e., abdominal distension, nausea) are likely to be managed in the outpatient setting. Moderate OHSS is characterized by the presence of ascites on imaging and laboratory studies notable for leukocytosis and hemoconcentration. Severe OHSS includes additional clinical symptoms (dyspnea, pleural effusion, oliguria), laboratory abnormalities (evidence of renal impairment, hyponatremia, and hyperkalemia), and worsened hemoconcentration and leukocytosis.^1^

A clear understanding of the pathophysiology of OHSS is important to identify and treat its clinical and laboratory features. Ovarian hyperstimulation leads to the release of vasoactive factors that contribute to capillary leakage and third spacing. This leads to tissue edema as well as hypovolemia. A prior publication in *MedEdPORTAL,* which complements the current resource, discusses the pathophysiology of OHSS and the contributing hormones.^5^ Our resource is unique in applying the pathophysiology of OHSS to the clinical setting and challenging learners to astutely evaluate, diagnose, and treat an undifferentiated emergency department patient with OHSS.

As the use of ART continues to increase, it is important that emergency medicine (EM) residents receive training in the diagnosis and management of OHSS. Our residents train in institutions with robust reproductive endocrinology departments, so some have seen a case of OHSS during their training. While moderate/severe OHSS is rare, EM residents should be exposed to the diagnosis, as we expose them to many other rare but potentially life-threatening diagnoses during their training. Several of the symptoms of OHSS can mimic other life-threatening conditions (e.g., pulmonary embolism). Simulation provides the optimal active learning environment to teach this topic and highlights many key principles important in the care of any unstable patient. This case was developed to target EM residents of all levels, recognizing that more senior residents would have an increased likelihood of having encountered a case of OHSS in clinical care.

## Methods

### Development

The Harvard affiliated EM residency incorporated a robust simulation curriculum as a core aspect of its didactics program. Each session was assigned an overarching topic and theme and included a combination of mannequin-based simulation cases, procedure practice stations, and small-group activities. While the list of procedural topics remained relatively constant, the topics for the mannequin-based cases were frequently rotated. Cases were developed using the backwards design model, by which the faculty and residency leadership initially identified the desired objectives.^6^ Then, the cases were designed to achieve the objectives.

This simulation was designed by EM faculty members with both clinical and academic interests in reproductive health, medical simulation, and medical education to address an identified need. After the objectives had been determined, the case, including the initial history and physical examination as well as the flow of the case, was designed. Additionally, we identified the necessary supplies and stimuli to create a realistic experience.

The simulation was performed in the STRATUS Center for Medical Simulation at Brigham and Women's Hospital during the Harvard affiliated EM obstetrics and gynecology session (Appendix A). The learners were PGY 1–4 EM residents. An EM faculty member facilitated the simulation. The project was undertaken as a quality improvement initiative at Brigham and Women's Hospital, and as such, it was not formally supervised by the institutional review board, per their policies.

### Equipment/Environment

We recommend the following equipment to implement this simulation case:

• High-fidelity female mannequin (low-fidelity mannequin can be used)
• Noninvasive blood pressure cuff
• Pulse oximeter
• Monitor that can be connected to the simulator to display the heart rhythm, heart rate, pulse oximetry, respiratory rate, and blood pressure
• Nasal cannula oxygen tubing
• Intravenous line start supplies
• Intravenous fluids
• Chest radiography with bilateral pleural effusions (Appendix B)
• EKG stimulus (Appendix B)
• Lab results sheet (Appendix B)
• Ultrasound simulator with radio-frequency identification chips loaded (ultrasound images can also be projected on a computer)
• Ultrasound video clips demonstrating enlarged ovaries and pelvic free fluid (Appendix C), free fluid in right upper quadrant and pleural effusion (Appendix D), and free fluid in left upper quadrant (Appendix E)

### Personnel

During each simulation, the facilitator divided the learners into groups of approximately four EM residents working as a team during the scenario. An EM attending physician facilitated the simulation, serving as the voice of the patient and providing relevant history and physical exam findings when asked. A simulation technician was responsible for changing the vital signs during the simulation based on the actions taken by the team as well as for projecting radiology studies (e.g., chest X-ray).

### Implementation

Within the simulation center, learners participated in the simulation in our human patient simulation room, which was set up to reflect a typical room in our emergency department. The residents entered the room, and the facilitator told them that they would be caring for a 28-year-old female patient who was presenting to the emergency department with shortness of breath and near syncope. After setting the stage for the scenario, the facilitator left the room and sat with the simulation specialist behind a one-way mirror. The facilitator used an intercom system to communicate with the learners and provide history as the patient. After a chest X-ray had been requested, the image (Appendix B) was projected onto the monitor for the learners to review and interpret. We embedded the ultrasound video clips (Appendices C–E) on chips attached to the mannequin. When the ultrasound probe scanned over the appropriate chip, the clips of the ultrasound videos displayed for the learners to view and interpret. Learners used a phone that rang in the control room to call consults. The facilitator answered the phone and played the role of the consultant. Based on actions taken by the learners, the patient's vital signs changed as the scenario progressed.

During the scenario, the facilitator made note of the critical actions (Appendix F performed (or missed) by the learners and reviewed them during the debriefing.

### Debriefing

Following completion of the case scenario, the facilitator entered the room to begin the debriefing using the debriefing materials (Appendix G). We preferred to start with an open-ended question such as “How did that feel?” as this gave learners permission to share their self-reflections of the experience. This allowed for both knowledge-based reflections (e.g., “I wasn't sure how to interpret the ultrasound image”) and emotional reflections (e.g., “I felt uncomfortable when the patient became hypotensive, and I wasn't sure what to do”). By beginning with an open-ended question, the debriefing could occur in the form of a rich discussion as opposed to simply going through the critical actions and noting which ones had been achieved or missed. Following general reflections, the debriefing also included a discussion about the identification and treatment of OHSS.

### Assessment

EM education faculty developed a postsimulation survey (Appendix H) that was distributed to residents at the completion of the debriefing to assess both their previous knowledge of OHSS and their experience caring for a patient with OHSS. This survey evaluated several of the principles of the Michigan Standard Simulation Experience Scale.^7^ We asked residents about both their prior knowledge of OHSS and whether they had previously cared for a patient with OHSS. Residents rated their comfort managing a patient with OHSS prior to and following the simulation on a 5-point Likert scale (1 = *not at all comfortable,* 3 = *somewhat comfortable,* 5 = *extremely comfortable*). We also asked residents about their agreement with a statement that the ultrasound clips enhanced the educational value of the case scenario, based on a 5-point Likert scale (1 = *strongly disagree,* 5 = *strongly agree*). In addition, learners were asked for narrative comments about the simulation scenario.

## Results

A total of 24 EM residents participated in this simulation. With regard to postgraduate year, 10 were first-year residents, five were second-year residents, six were third-year residents, and three were fourth-year residents.

Fifteen residents (62%) reported having heard of OHSS prior to the simulation, seven residents (29%) had not heard of OHSS, and two residents (8%) were unsure. Four residents (17%) reported having previously cared for a patient with OHSS, while the remainder had not.

With regard to their comfort managing a patient with OHSS, learners reported their prescenario comfort at a mean of 1.7 on a 5-point Likert scale (1 = *not at all comfortable,* 5 = *extremely comfortable*). Postscenario, learners’ comfort increased to a mean of 3.7 (*p* < .001). Learners felt that the ultrasound video clips added value to the scenario (mean score of 4.6 on 5-point Likert scale).

Eight residents provided narrative comments, all of which were positive. Examples of comments include the following: “Good case, lends itself well to multiple differential diagnoses,” “Ultrasound was very beneficial to learning,” and “Great job with complex management.”

## Discussion

The development of this simulation on OHSS addressed a potential gap in training for EM residents. As reported by our group of learners, 83% had never cared for a patient with OHSS in the emergency department and had limited confidence in their ability to do so prior to participating in this simulation. While previous publications have described the pathophysiology and treatment of OHSS,^1,3,8,9^ this simulation provided a unique opportunity for active learning. Our learners reported a significantly increased level of comfort managing OHSS after the simulation.

There is limited literature on training EM residents in the diagnosis and management of OHSS. One publication focuses on utilizing self-directed learning and the flipped classroom to teach topics in OB/GYN emergencies including OHSS to EM residents,^10^ but to our knowledge, ours is the first to utilize simulation in this effort. Simulation can be particularly useful as residents are able to work in an environment that mimics their clinical practice. In addition, this simulation pushes residents to consider a broad differential diagnosis and highlights the importance of utilizing point-of-care ultrasound in the care of emergency department patients.

### Lessons Learned

Reflecting on the implementation of this simulation, it is important to consider that emergency physicians have varying degrees of knowledge of OHSS. Faculty development and education are important to ensure optimal learning for the resident trainees. Our debriefing materials and references can be utilized by facilitators to refamiliarize themselves with OHSS prior to leading the simulation for trainees.

We had mixed groups of resident trainees for this simulation, ranging from PGY 1 to PGY 4. This worked well for the case scenario, as our more senior residents were more likely to have seen a case of OHSS during their clinical training. In addition, our senior residents were more proficient in point-of-care ultrasound and could offer peer coaching to their more junior colleagues during the simulation. While we included only EM residents in this simulation experience, the case would also be an excellent opportunity for interdepartmental training with OB/GYN residents.

### Limitations

A limitation of this resource is the use of high-fidelity simulation to make the scenario as close to our actual emergency department experience as possible. High-fidelity simulation allowed us to place our learners in an environment closely mimicking their actual practice environment. However, the simulation could be easily adapted to an environment with fewer resources. The facilitator could provide information including vital signs and could verbally acknowledge actions performed by the team that may not be possible on a low-fidelity mannequin (i.e., intravenous line placement). This case could also be used in an oral boards–style format if no mannequin is available.

As our residents had received training in point-of-care ultrasound, many of them reached for it early in their patient evaluations to aid in diagnosis and treatment. Because we embedded the ultrasound clips on chips attached to the mannequin, they were able to be read by the ultrasound probe when applied to the correct area on the mannequin and displayed for the learners. While this provided an added level of reality, the simulation can be used by educators without access to this technology. The ultrasound clips can be displayed on a computer monitor for interpretation, or a verbal description of the findings can be provided to the learners if video display is not available.

Another limitation is in the curriculum's assessment, which relied on learners’ self-reported confidence in managing OHSS as opposed to objective observation in a follow-up scenario after the training. Learner confidence has limitations as a measurement of competence. We used the critical action checklist as a guide for the facilitator during the scenario and to assist with debriefing, but we did not record or analyze the number of critical actions achieved by each group of learners. Our critical action checklist could be used in this capacity. In addition, to avoid revealing the case diagnosis ahead of the simulation, questions assessing residents’ self-perception of their knowledge of OHSS and comfort managing a patient with OHSS prior to the simulation were included on the postsimulation survey. This could have introduced both response bias and order effect bias in our results.

### Conclusion

In summary, OHSS is a complication of ART that EM physicians are likely to encounter in clinical practice, as the number of people using this technology continues to increase. This simulation provides an excellent opportunity to explore the symptoms, clinical and laboratory manifestations, and treatment options for OHSS. The value of the simulation was highlighted by our learners. While the simulation was limited to EM residents, it could also be applicable for OB/GYN residents and physician assistants who work in an emergency department setting.

## Appendices


OHSS Simulation.docxSimulation Labs, Chest X-ray, & EKG.docxUS Clip - Pelvis.mp4US Clip - RUQ.mp4US Clip - LUQ.mp4Critical Actions.docxDebriefing Materials.docxOHSS Survey.docx
*All appendices are peer reviewed as integral parts of the Original Publication.*
