# Supplementary material for: Ovarian Hyperstimulation Syndrome: A Simulation Case for Emergency Medicine Residents
Source: MedEdPORTAL. 2022 Sep 6;18:11271. doi: 10.15766/mep_2374-8265.11271 (PMC9445087; doi:10.15766/mep_2374-8265.11271)

Appendix B: Laboratory results, chest x-ray and EKG

CBC

WBC: 19.0 10^3^ uL^-1^ (ref 4.5-11 10^3^ uL^-1^)

Hct: 49.2% (ref: 35-45%)

Hgb: 16.4 g/dL (ref: 12-15.5 g/dL)

Platelets: 169 10^3^ uL^-1^ (ref: 150-450 10^3^ uL^-1^)

Chemistries

Na: 128 mEq/L (ref: 136-142 mEq/L)

K: 5.2 mEq/L (ref: 3.5-5 mEq/L)

Cl: 95 mEq/L (ref 96-106 mEq/L)

CO2: 22 mEq/L (ref 22-28 mEq/L)

BUN: 40 mg/dL (ref : 8-23 mg/dL)

Cr: 1.89 mg/dL (ref : 0.3-0.9 mg/dL)

Glu: 89 mg/dL (ref : 70-110 mg/dL)

AST 54 U/L (ref : 20-48 U/L)

ALT 45 U/L (ref : 10-40 U/L)

Alkaline phosphatase 124 (ref 50-120 U/L)

Total bilirubin 1.1 mg/dL (ref: 0.3-1.2 mg/dL)

Lipase 35 U/L (ref: 0-160 U/L)

Urinalysis

Normal

Urine hCG

Positive

Serum HCG

47 IU/L (ref : <3 IU/L)

D-dimer

779 ng/mL (ref : <500 ng/mL)

Coagulation

PT 12.2 sec (ref: 10-13 sec)

PTT 30.6 sec (ref: 25-40 sec)

Chest x-ray demonstrating right greater than left pleural effusions (Author Owned)


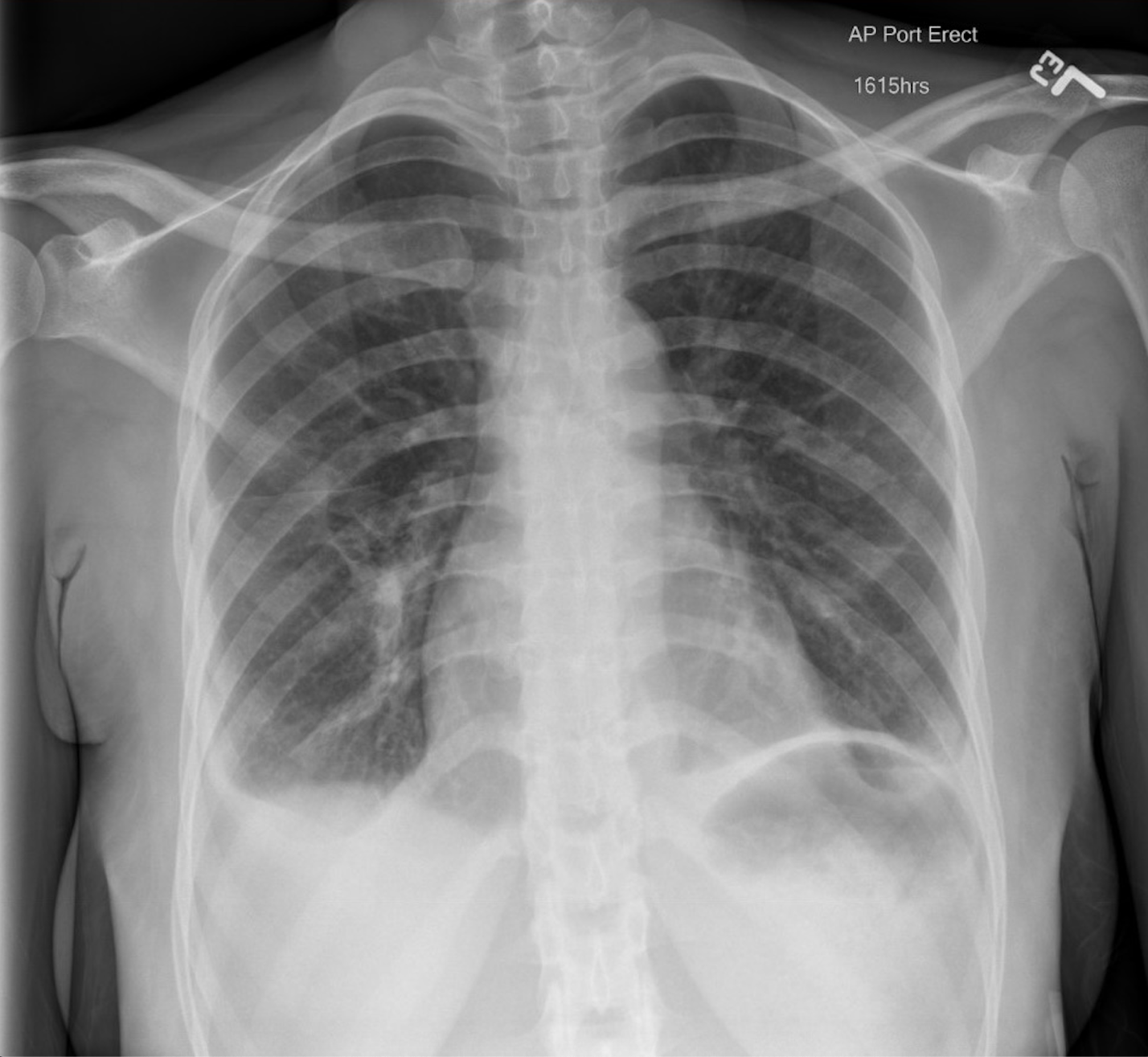


EKG: sinus tachycardia, rate 110, normal intervals, no ST segment abnormalities

(Author owned)


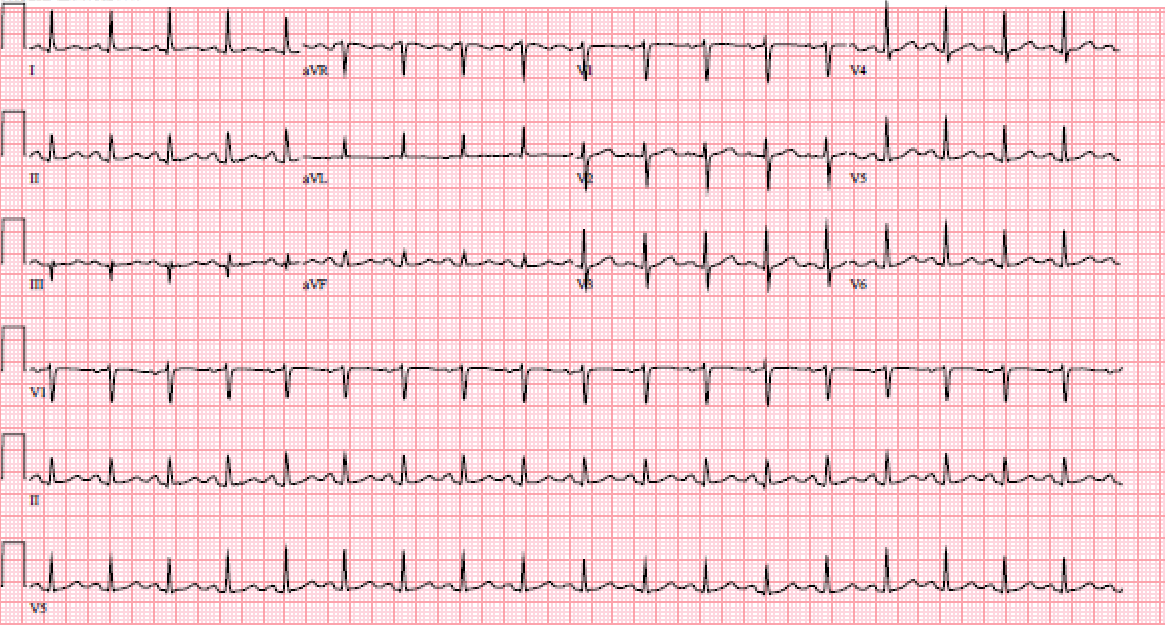

Supplement: Supplementary file 1 — OHSS Simulation.docxSimulation Labs, Chest X-ray, & EKG.docxUS Clip - Pelvis.mp4US Clip - RUQ.mp4US Clip - LUQ.mp4Critical Actions.docxDebriefing Materials.docxOHSS Survey.docx [file mep_2374-8265.11271-s001.zip › B. Simulation Labs, CXR, & EKG.docx]
