# Supplementary material for: Ovarian Hyperstimulation Syndrome: A Simulation Case for Emergency Medicine Residents
Source: MedEdPORTAL. 2022 Sep 6;18:11271. doi: 10.15766/mep_2374-8265.11271 (PMC9445087; doi:10.15766/mep_2374-8265.11271)
Supplement: Supplementary file 1 — OHSS Simulation.docxSimulation Labs, Chest X-ray, & EKG.docxUS Clip - Pelvis.mp4US Clip - RUQ.mp4US Clip - LUQ.mp4Critical Actions.docxDebriefing Materials.docxOHSS Survey.docx [file mep_2374-8265.11271-s001.zip › F. Critical Actions.docx]

Appendix F. OHSS Simulation Case – Critical Actions Checklist

|  | Done correctly | Done incorrectly | Not done |
| --- | --- | --- | --- |
| Obtain a focused history |  |  |  |
| Perform a focused physical examination |  |  |  |
| Obtain intravenous access |  |  |  |
| Give intravenous fluids |  |  |  |
| Initiate supplemental oxygen therapy |  |  |  |
| Perform bedside ultrasound |  |  |  |
| Recognize enlarged ovaries and free fluid |  |  |  |
| Recognize diagnosis of OHSS |  |  |  |
| Consult gynecology |  |  |  |
| Admit patient to the hospital |  |  |  |

Anchor examples for critical actions checklist

|  | Done correctly | Done incorrectly |
| --- | --- | --- |
| Obtain a focused history | Efficiently obtains historical information from patient including recent fertility treatment | Obtains some information from patient but misses key details such as recent fertility treatment |
| Perform a focused physical examination | Identifies abnormal pulmonary, cardiovascular and abdominal examination findings. | Misses some abnormal exam findings. |
| Obtain intravenous access | Quickly identifies patient acuity and obtains IV access | Delay in obtaining IV access |
| Give intravenous fluids | Immediately initiates IVF after IV access obtained | Delay in initiating IVF |
| Initiate supplemental oxygen therapy | Recognizes hypoxia and immediately initiates oxygen therapy | Delay in initiating supplemental oxygen therapy |
| Perform bedside ultrasound | Following initial history and examination, learner requests and performs bedside ultrasound | Delay in performing bedside ultrasound or does not obtain all images |
| Recognize enlarged ovaries and free fluid | Verbalizes the abnormal findings, including enlarged ovaries and abdominal free fluid | Incompletely interprets images (i.e. recognizes free fluid but not enlarged ovaries) |
| Recognize diagnosis of OHSS | Verbalizes OHSS as the likely etiology of this patient’s symptoms | Does not initially consider OHSS as a possible diagnosis but responds to data and facilitator prompts |
| Consult gynecology | Consults gynecology early in the patient’s course once there is suspicion of OHSS | Delay in gynecology consultation |
| Admit patient to the hospital | Requests that the patient be admitted to the gynecology service. | n/a |
