# Supplementary material for: Ovarian Hyperstimulation Syndrome: A Simulation Case for Emergency Medicine Residents
Source: MedEdPORTAL. 2022 Sep 6;18:11271. doi: 10.15766/mep_2374-8265.11271 (PMC9445087; doi:10.15766/mep_2374-8265.11271)
Supplement: Supplementary file 1 — OHSS Simulation.docxSimulation Labs, Chest X-ray, & EKG.docxUS Clip - Pelvis.mp4US Clip - RUQ.mp4US Clip - LUQ.mp4Critical Actions.docxDebriefing Materials.docxOHSS Survey.docx [file mep_2374-8265.11271-s001.zip › G. Debriefing Materials.docx]

**Appendix D: Debriefing Materials**

Flow of debriefing:

Following completion of the case scenario, the facilitator enters the room to begin the debriefing. We prefer to start with an open-ended question to begin, such as “How did that feel?”. Beginning with this question gives the learner permission to share their self-reflections of the experience, allowing for both knowledge-based reflections (i.e., “I wasn’t sure how to interpret the ultrasound image”) as well as emotional reflections (i.e., “I felt uncomfortable when the patient became hypotensive, and I wasn’t sure what to do.”). By beginning with an open-ended question, it allows the debriefing to occur in the form of a rich discussion as opposed to simply going through the critical actions and noting which ones were achieved or missed. If the learners do not share reflections when asked “How did that feel?”, follow-up questions can include the following:

- What do you think went well?
- What did you struggle with?
- What was your thought process when you chose (fill in example) intervention?

Following general reflections, the debriefing should also include a discussion about the identification and treatment of OHSS. Questions for learner reflection include the following:

- What are the risk factors for OHSS?
- What is the pathophysiology of OHSS?
- What would you expect to see in the clinical presentation of a patient with OHSS?
- How do you classify OHSS as mild, moderate, and severe?
- What diagnostic modalities are available to assist with the diagnosis?
- What are your treatment priorities in suspected OHSS?

Content resources for facilitators

Facilitators who are unfamiliar with OHSS should consider topical review prior to teaching this simulation case.

A prior MedEdPortal publication focuses on the details of the pathophysiology of OHSS:

- Zachow R. Applied and Integrative Endocrinology Case: A 31-Year-Old Woman with Shortness of Breath, Chest Pain, Nausea, and Dizziness. MedEdPortal.2012.

Below, we include a review of clinical information regarding OHSS. We used the following sources and recommend them for further reading:

- Timmons, D., Montrief, T., Koyfman, A., & Long, B. (2019). Ovarian hyperstimulation syndrome: a review for emergency clinicians. *The American journal of emergency medicine*, *37*(8), 1577-1584.
- Practice Committee of the American Society for Reproductive Medicine. (2008). Ovarian hyperstimulation syndrome. *Fertility and sterility*, *90*(5), S188-S193.
- Kumar, P., Sait, S. F., Sharma, A., & Kumar, M. (2011). Ovarian hyperstimulation syndrome. *Journal of human reproductive sciences*, *4*(2), 70.
- Budev, M. M., Arroliga, A. C., & Falcone, T. (2005). Ovarian hyperstimulation syndrome. *Critical care medicine*, *33*(10), S301-S306.
- Blumenfeld, Z. (2018). The ovarian hyperstimulation syndrome. *Vitamins and hormones*, *107*, 423-451.

**Risk factors for OHSS**:

- Age < 35
- Low body weight
- History of polycystic ovarian syndrome
- History of OHSS
- Higher doses of exogenous gonadotropins
- Increased number of follicles
- Elevated serum estradiol and rate of estradiol rise
- Pregnancy

**Pathophysiology**:

- Ovarian stimulation causes overproduction of pro-inflammatory and vasoactive cytokines leading to increased capillary permeability
- hCG (used as an ovulatory trigger in ART) increases vascular endothelial growth factor which increases vascular permeability
- Arteriolar vasodilation and increased capillary permeability leads to fluid shifting to the extravascular space
- Increased follicular fluid levels of prorenin and renin
- Levels of vascular endothelial growth factor (VEGF), an angiogenic cytokine, have been linked to severity of OHSS

**Clinical Presentation:**

- Abdominal distension and pain
- Nausea/vomiting/diarrhea
- Increased intra-abdominal pressure can cause organ dysfunction
  - Oliguria
  - Acute kidney injury
  - Hepatic injury
  - Bowel edema (leading to vomiting, diarrhea)
  - Hyponatremia
- Hemoconcentration
- Dyspnea, which may be related to ascites or pulmonary edema in severe disease
- Thrombosis/hypercoagulability

**OHSS Classification**:

- Mild: Abdominal distension, mild nausea, vomiting, diarrhea, ovarian enlargement
  - Grade 1 - Abdominal distention and discomfort
  - Grade 2 - Grade 1 disease plus nausea, vomiting and/or diarrhea plus ovarian enlargement from 5 to 12 cm
- Moderate: Ascites present on imaging
  - Grade 3 - Features of mild OHSS plus ultrasonographic evidence of ascites
- Severe: Ascites appreciated on examination, pleural effusion, intractable nausea and vomiting, dyspnea, oliguria
  - Grade 4 - Features of moderate OHSS plus clinical evidence of ascites and/or pleural effusion and breathing difficulties
  - Grade 5 - All of the above plus a change in the blood volume, increased blood viscosity due to hemoconcentration, coagulation abnormalities and diminished renal perfusion and function

**Diagnostic modalities**:

- Laboratory studies to evaluate for end organ injury and electrolyte abnormalities. Common abnormalities in serious illness include:
  - Hemoconcentration
  - Leukocytosis
  - Hyponatremia
  - Hyperkalemia
  - Elevated LFTs
  - Elevated creatinine
- hCG (as pregnancy is a risk factor for OHSS)
- Type and screen (if concern for possible hemorrhage)
- Bedside ultrasound – can identify enlarged ovaries, ascites, pleural effusion. Ultrasound is also helpful to rule out other diagnoses (i.e. ectopic pregnancy). Evaluation of the IVC can help with assessment of intravascular volume status.
- Chest x-ray – identify pleural effusions

**Approach to Management:**

- Frequent vital signs, including daily weights
- Strict fluid management: maintain circulatory hemodynamics with crystalloid (consider albumin for severe hemoconcentration, severe hypoalbuminemia, or severe ascites)
- Monitor urine output (goal > 20-30 mL/h). Use diuretic agents if necessary, after intravascular volume has been restored.
- Correct electrolyte abnormalities (consider hypertonic saline for hyponatremia)
- Thoracentesis if needed to support ventilation
- Consider paracentesis for severe OHSS but recognize that large volume paracentesis may lead to rapid re-accumulation of fluid. Ultrasound guidance is important to avoid puncturing a large ovarian cyst.
- Avoid bimanual examination of the ovaries due to risk of ovarian rupture
