# Supplementary material for: Ovarian Hyperstimulation Syndrome: A Simulation Case for Emergency Medicine Residents
Source: MedEdPORTAL. 2022 Sep 6;18:11271. doi: 10.15766/mep_2374-8265.11271 (PMC9445087; doi:10.15766/mep_2374-8265.11271)
Supplement: Supplementary file 1 — OHSS Simulation.docxSimulation Labs, Chest X-ray, & EKG.docxUS Clip - Pelvis.mp4US Clip - RUQ.mp4US Clip - LUQ.mp4Critical Actions.docxDebriefing Materials.docxOHSS Survey.docx [file mep_2374-8265.11271-s001.zip › H. OHSS Survey.docx]

**Appendix H: Ovarian Hyperstimulation Syndrome Survey**

1. What is year in training are you? (Circle one) PGY1 PGY2 PGY3 PGY4
2. Prior to this session, had you ever heard of Ovarian Hyperstimulation Syndrome? Please circle one?

Yes No Unsure

1. Prior to this session, had you ever managed a patient with Ovarian Hyperstimulation Syndrome? Please circle one

Yes No Unsure

1. Using the scale below, please rate your comfort managing patients with Ovarian Hyperstimulation Syndrome **PRIOR TO THIS SESSION** (Circle one)

1 2 3 4 5

Not at all comfortable Somewhat comfortable Extremely comfortable

1. Using the scale below, please rate your comfort managing patients with Ovarian Hyperstimulation Syndrome **AFTER THIS SESSION** (Circle one)

1 2 3 4 5

Not at all comfortable Somewhat comfortable Extremely comfortable

1. The ultrasound clips enhanced the educational value of the case scenario

1 2 3 4 5

Strongly disagree Disagree Neutral Agree Strongly agree

Comments:___________________________________________________________________________________________________________________________________________________________________________________________________________________________________________________________________________________________________________________________________________
