# Supplementary material for: Ovarian Hyperstimulation Syndrome: A Simulation Case for Emergency Medicine Residents
Source: MedEdPORTAL. 2022 Sep 6;18:11271. doi: 10.15766/mep_2374-8265.11271 (PMC9445087; doi:10.15766/mep_2374-8265.11271)
Supplement: Supplementary file 1 — OHSS Simulation.docxSimulation Labs, Chest X-ray, & EKG.docxUS Clip - Pelvis.mp4US Clip - RUQ.mp4US Clip - LUQ.mp4Critical Actions.docxDebriefing Materials.docxOHSS Survey.docx [file mep_2374-8265.11271-s001.zip › A. OHSS Simulation.docx]

| **Appendix A**  **SIMULATION CASE TITLE: Ovarian Hyperstimulation Syndrome (OHSS)**  **AUTHORS: Kathleen Wittels, MD, Katherine Dickerson Mayes, MD, PhD, Andrew Eyre, MD, MSHPEd** | |
| --- | --- |
| **PATIENT NAME: Rebecca Jones**  **PATIENT AGE: 28**  **CHIEF COMPLAINT: Shortness of breath and near-syncope** | |
|  | |
| **Brief narrative description of case** | 28-year-old female who is undergoing treatment for infertility presents with 5 days of progressively worsening shortness of breath and an episode of near syncope. Learners will be expected to obtain a pertinent history (including recent infertility treatment), diagnose OHSS, and provide appropriate supportive care. |
| **Primary Learning Objectives** | 1. Verbalize the common signs and symptoms of OHSS 2. Identify OHSS using common laboratory and radiologic tests 3. Demonstrate appropriate supportive care for OHSS (intravenous fluids, oxygen, admission) |
| **Critical Actions** | 1. Obtain a focused history, including recent infertility treatment 2. Perform focused physical examination 3. Obtain IV access and start intravenous fluid resuscitation 4. Initiate supplemental oxygen therapy 5. Perform pelvic ultrasound and recognize enlarged ovaries and free fluid 6. Recognize diagnosis of OHSS 7. Consult gynecology 8. Admit the patient to the hospital for ongoing care |
| **Learner Preparation** | You are working in the Emergency Department and are called to see a 28-year-old female patient who is presenting with 5 days of progressively worsening shortness of breath. She presented today due to an episode of near syncope after getting up from bed. You have access to standard radiology services (x-ray, CT, ultrasound, and MRI), laboratory services, and subspecialty consultations. |

| Initial Presentation | | | |
| --- | --- | --- | --- |
| **Initial vital signs** | Temp: 98.8°F HR: 115 BP: 95/55 RR: 22 SpO2: 93% on room air Weight: 79kg | | |
| **Overall Appearance** | 28-year-old female patient who is seated on the Emergency Department stretcher with moderately increased work of breathing. | | |
| **Actors and roles in the room at case start** | Patient: High-Fidelity Mannequin with voice provided from outside room. Patient can be played by faculty, simulation specialist, or standardized patient. Low fidelity mannequin or standardized patient can replace high fidelity mannequin if needed.  Care Team: Care team can be divided into clinician and nursing roles, played by residents, medical students, physician assistants, or other interprofessional learners.  Embedded Actors: None | | |
| **HPI** | 28-year-old female presents to the emergency department with 5 days of progressively worsening shortness of breath. She presented today due to an episode of near syncope after getting up from bed.  Additional history if asked:  -The shortness of breath is worse when supine  -She has some associated abdominal pain and bloating as well as nausea and non-bloody diarrhea.  -She had no chest pain, vomiting, vaginal bleeding, vaginal discharge, dysuria, or hematuria  -She has gained 10-15 pounds over the last week  -She is seeing a fertility specialist due to inability to conceive over the last 2 years | | |
| **Past Medical/Surgical History** | **Medications** | **Allergies** | **Family History** |
| Polycystic Ovarian Syndrome  Appendectomy | FSH and HCG (recently started) | Penicillin (Rash) | Noncontributory |
| **Physical Examination** | | | |
| **General** | Appears stated age, sitting on stretcher, moderately increased work of breathing | | |
| **HEENT** | No abnormalities | | |
| **Neck** | No abnormalities | | |
| **Lungs** | Decreased breath sounds bilaterally. Increased work of breathing. | | |
| **Cardiovascular** | Tachycardic, regular rate and rhythm, no murmurs | | |
| **Abdomen** | Moderate abdominal distension. Mild diffuse tenderness to palpation. | | |
| **Neurological** | Normal speech, cranial nerves, strength, and sensation. | | |
| **Skin** | No abnormalities | | |
| **GU** | Normal external genitalia. No vaginal discharge. No cervical motion tenderness, mild bilateral adnexal tenderness. No costovertebral angle tenderness. | | |
| **Psychiatric** | No abnormalities | | |

| Instructor Notes - Changes and CASE Branch Points | | |
| --- | --- | --- |
| **Intervention / Time point** | **Change in Case** | **Additional Information** |
| *3 minutes into case* | If IV fluids administered, HR drops to 105 and BP 105/78  If no IV fluids started, HR rises to 122 and BP drops to 88/52  If oxygen applied, SpO2 increases to 97%  If no oxygen applied, SpO2 decreases to 91% |  |
| *5 minutes into case* | EKG is done (if requested): Shows sinus tachycardia  Chest x-ray is done (if requested): Shows bilateral pleural effusions  Bedside ultrasound is performed (if requested): shows enlarged ovaries, free fluid, and pleural effusion | If no oxygen started, patient will complain of worsening shortness of breath.  If learners cannot interpret ultrasound images, radiology can provide the formal read. |
| *10 minutes into case* | Labs (if ordered) are available and presented to team demonstrating hyponatremia, hyperkalemia, elevated creatinine, elevated hematocrit, elevated d-dimer | If team has not obtained infertility history, patient will ask “Can someone call my infertility doctor?” |
| *15 minutes into case* | If team consults gynecology or discusses with the infertility specialist, they will admit the patient but cannot come immediately to see her as they are starting a c-section. | If elevated d-dimer not recognized, lab control will call the room with a “critical value.”  If team has not considered OHSS as diagnosis, patient will ask “could my infertility injections be causing this?”  If team orders a chest CT scan to evaluate for a pulmonary embolus, radiology says they cannot do it given the elevated creatinine.  If team has not considered OHSS, the patient’s infertility specialist can call to discuss the patient’s presentation. |
| 20 minutes into case | Case Complete |  |

**Ideal Scenario Flow**

The learners enter the room to find the patient sitting upright with increased work of breathing. They immediately place the patient on bedside monitors and recognize that the patient is tachycardic, hypotensive, and somewhat hypoxic. They place the patient on oxygen, obtain IV access, and order a bolus of normal saline with improvement in vital signs. They obtain a history of present illness, including the patient’s history of infertility and recent hormone therapy. After performing a focused physical examination, the learners will consider the differential diagnosis including OHSS, pneumonia, ectopic pregnancy, or ruptured ovarian cyst. They will order a chest x-ray that demonstrates bilateral pleural effusions. They will obtain labs that demonstrate hyperkalemia, hyponatremia, elevated creatinine, elevated hematocrit, elevated d-dimer, and positive HCG (with low quantitative level). They will either perform or order a pelvic ultrasound demonstrating enlarged ovaries and free fluid. Based on the history of infertility treatment, ascites, ovarian enlargement, and pleural effusions, the learners will make the diagnosis of OHSS, or at least consider this in the differential. Although the d-dimer is elevated, the learners will recognize that there is an alternative diagnosis that better explains the symptoms and thus a CT chest pulmonary angiogram is not necessary. The learners will consult gynecology, continue supportive care, and admit the patient to the hospital.

**Anticipated Management Mistakes**

1. Failure to recognize ability to perform ultrasound: We utilized a portable ultrasound simulator with radio-frequency ID cards that had been loaded with the appropriate images. During the case pre-brief, learners were reminded that ultrasound and specialty consultations were available.
2. Failure to correctly interpret ultrasound: Some of our learners had difficulty interpreting the ultrasound images. During the case, radiology can be consulted to assist with interpretation. However, discussion of the ultrasound images should be included in the case debriefing.
3. Unfamiliarity with the diagnosis: Some of our learners had little or no knowledge of ovarian hyperstimulation syndrome. We ran this case with learners of mixed levels, thus increasing the likelihood that some members of the team had some experience with this process. Prompts can be provided by the patient or additional guidance can be provided if the team consults gynecology. An overview of this syndrome should be covered in the debrief.
